# Compliance increase in 189 units participating in the German hand hygiene campaign “AKTION Saubere Hände”

**DOI:** 10.1186/1753-6561-5-S6-O62

**Published:** 2011-06-29

**Authors:** C Reichardt, K Bunte-Schönberger, P van der Linden, J Clausmeyer, F Schwab, P Gastmeier, M Behnke

**Affiliations:** 1 Institute of Hygiene and Environmental Medicine, University Medicine Berlin - Charite, Berlin, Germany

## Introduction / objectives

The national German hand hygiene (HH) campaign “AKTION Saubere Hände” started at January 1^st^ 2008. The campaign is based on the WHO “Clean Care is Safer Care” campaign. By March 30^th^ 2011, over 800 health care institutions are actively participating. Among other measures, hand hygiene compliance observations are a voluntary part of the campaign.

We present compliance rates before and after intervention in 189 units in 43 hospitals.

## Methods

All participants used defined observation tools. The definition of HH opportunities (HHO) is based on the WHO Model “My 5 moments of hand hygiene”. Observations were done before and after intervention. A minimum of 200 observations per unit and 20 observations per HHO was defined. Results were stratified by HHO, type of HCW and type of unit. Change of compliance was analysed by Wilcoxon and Kruskal Wallis test.

## Results

75.391 HHO‘s were observed in 189 units in 43 hospitals. There was a significant median increase of compliance in all HHO’s (p<0.001) as well as in all unit types (6% internal med., 11% interdisciplinary, 8% Surgery, 21% pediatric, 17% neonatology). Physicians improved by 14% as well as nurses (p<0.001>), nurse students by 15% and other HCW by 10% (p<0.001). There was no increase in Medical students.

## Conclusion

Hospitals participating in the campaign have to implement a package of interventions. Compliance observations are an option. These 43 hospitals contain a variety of hospitals from large tertiary care to small (more than 800 and less than 400 beds) primary care hospitals. Our results show, that our multimodal intervention model lead to compliance improvements in different types of settings.

## Disclosure of interest

None declared.

